# Ethics and Law in Research on Algorithmic and Data-Driven Technology in Mental Health Care: Scoping Review

**DOI:** 10.2196/24668

**Published:** 2021-06-10

**Authors:** Piers Gooding, Timothy Kariotis

**Affiliations:** 1 Melbourne Law School University of Melbourne Melbourne Australia; 2 Mozilla Foundation Mountain View, CA United States; 3 Melbourne School of Government University of Melbourne Melbourne Australia

**Keywords:** digital psychiatry, digital mental health, machine learning, algorithmic technology, data-driven technology, artificial intelligence, ethics, regulation, law, mobile phone

## Abstract

**Background:**

Uncertainty surrounds the ethical and legal implications of algorithmic and data-driven technologies in the mental health context, including technologies characterized as artificial intelligence, machine learning, deep learning, and other forms of automation.

**Objective:**

This study aims to survey empirical scholarly literature on the application of algorithmic and data-driven technologies in mental health initiatives to identify the legal and ethical issues that have been raised.

**Methods:**

We searched for peer-reviewed empirical studies on the application of algorithmic technologies in mental health care in the Scopus, Embase, and Association for Computing Machinery databases. A total of 1078 relevant peer-reviewed applied studies were identified, which were narrowed to 132 empirical research papers for review based on selection criteria. Conventional content analysis was undertaken to address our aims, and this was supplemented by a keyword-in-context analysis.

**Results:**

We grouped the findings into the following five categories of technology: social media (53/132, 40.1%), smartphones (37/132, 28%), sensing technology (20/132, 15.1%), chatbots (5/132, 3.8%), and miscellaneous (17/132, 12.9%). Most initiatives were directed toward detection and diagnosis. Most papers discussed privacy, mainly in terms of respecting the privacy of research participants. There was relatively little discussion of privacy in this context. A small number of studies discussed ethics directly (10/132, 7.6%) and indirectly (10/132, 7.6%). Legal issues were not substantively discussed in any studies, although some legal issues were discussed in passing (7/132, 5.3%), such as the rights of user subjects and privacy law compliance.

**Conclusions:**

Ethical and legal issues tend to not be explicitly addressed in empirical studies on algorithmic and data-driven technologies in mental health initiatives. Scholars may have considered ethical or legal matters at the ethics committee or institutional review board stage. If so, this consideration seldom appears in published materials in applied research in any detail. The form itself of peer-reviewed papers that detail applied research in this field may well preclude a substantial focus on ethics and law. Regardless, we identified several concerns, including the near-complete lack of involvement of mental health service users, the scant consideration of algorithmic accountability, and the potential for overmedicalization and techno-solutionism. Most papers were published in the computer science field at the pilot or exploratory stages. Thus, these technologies could be appropriated into practice in rarely acknowledged ways, with serious legal and ethical implications.

## Introduction

### Background

Data-driven technologies for mental health have expanded in recent years [1,2]. The COVID-19 pandemic has accelerated this shift, with physical distancing measures fast-tracking the digitization and virtualization of health and social services [3,4]. These initiatives extend from hospital- to community-based services for people with mental health conditions and psychosocial disabilities (the term *mental health conditions and psychosocial disabilities* is used to refer to the broad range of mental health conditions and the associated disability; the term is used by the World Health Organization [5]). Government agencies, private technology firms, service user groups, service providers, pharmaceutical companies, professional associations, corporate services, and academic researchers are among the actors involved [5-7]. The technologies they create serve various functions, including information sharing, communication, clinical decision support, digital therapies, patient or service user and population monitoring, bioinformatics and personalized medicine, and service user health informatics [1]. Only some of these broader digital technologies will use algorithmic technologies to which this paper will turn.

Throughout this paper, we use the term algorithmic and data-driven technologies to describe various technologies that rely on complex information processing to analyze large amounts of personal data and other information deemed useful to making decisions [6]. The term is used here to encompass technologies variously referred to as artificial intelligence, machine learning, deep learning, natural language processing, robotics, speech processing, and similar automation technologies. The paper is premised on the view that the term *algorithmic and data-driven technologies* offers a useful category for the purposes of this review, although important conceptual and practical differences exist between technologies within this broad category (eg, between artificial intelligence and machine learning).

In the mental health context, algorithmic and data-driven technologies are generally used to make inferences, predictions, recommendations, or decisions about individuals and populations. Predictive analysis is largely aimed at assessing a person’s health conditions. Data collection may occur in a range of settings, from services concerning mental health, suicide prevention, or addiction support. Collection may also occur beyond these typical domains. For example, web-based platforms can draw on users’ posts or purchasing habits to flag their potential risk of suicide [7]. CCTV systems with machine learning sensors in *suicide hotspots* can be programmed to assess bodily movements that may precipitate a person’s suicide attempt [8]. Education institutions may flag students who appear to be in distress based on attendance records, social media use, and physiometric monitoring [9]. There are also examples of algorithmic technologies being used in forensic mental health settings [10] and other criminal justice settings [11], including databases that combine noncriminal mental health data with user-generated social media content for the apparent purpose of preventive policing [12].

Some prominent mental health professionals have argued that digital technologies, including algorithmic and data-driven technologies, hold the potential to bridge the “global mental health treatment gap” [13] by “reach[ing] billions of people” worldwide [14]. A 2019 *Lancet Psychiatry* editorial describes a “general agreement that big data and algorithms will help optimize performance in psychiatry” [15]. Others have described “widespread agreement by health care providers, medical associations, industry, and governments that automation using digital technology could improve the delivery and quality of care in psychiatry, and reduce costs” [16]. Indeed, governments and some private sector actors appear enthusiastic [1]. For people who use mental health services and their representative organizations, views on algorithmic technology in mental health care appear more ambivalent, although research by service user researchers, advocates, and their representative organizations comprises only a very small part of scholarship and commentary in the field [17-19].

This study set out to identify to what extent and on what matters legal and ethical issues were considered in the empirical research literature on algorithmic and data-driven technologies in mental health care. *Empirical research* refers simply to scholarship that seeks to use algorithmic and data-driven technology in an applied way in the mental health context.

### Ethics and Law

Ethics refer to guiding principles, whereas laws, which may be based on ethical or moral principles, are enforceable rules and regulations with penalties for those who violate them. Scholarship on the ethical and legal dimensions of algorithmic and data-driven technologies in mental health care is relatively scant but growing [20-26]. Existing research generally draws together two strands of research: first, the ethicolegal issues involved in algorithmic and data-driven technological mental health initiatives [25-27] and, second, a broader scholarship concerning algorithmic and data-driven technologies [28-30]. We briefly discuss each of these strands of research.

According to Lederman et al [25], most web-based mental health interventions have not been subject to ethical scrutiny, particularly those that go beyond one-to-one web-based or phone-based counseling, such as mental health apps and moderated web-based forums. Lederman et al [25] suggest using the classic health ethics framework, with its four principles of nonmaleficence, beneficence, respect for autonomy, and justice, particularly given its widespread use and acceptance among the health professions [26]. However, given the emergence of digital mental health initiatives in nonclinical settings (eg, in education, work settings, social media, and financial services), other ethical frameworks and practices may be required [7,31]. Nonhealth settings are not governed by the same entrenched bioethical principles, norms of conduct, or regulatory frameworks as formal health care systems [31]. Burr et al [31] pointed out that the transfer of responsibility from traditional health care providers to institutions, organizations (both private and public), and individuals who are creating web-based mental health initiatives gives rise to new ethical considerations. These include the duty to intervene in emergencies, competency to address people’s support needs, and ensuring the decisional capacity and health literacy of consumers of commercialized products [31]. This expanded scope is a sign that the ethical literature concerning digital technology in the mental health context is growing [20,24,32-34], even if ethical analyses may not occur in most applied initiatives, as suggested by Lederman et al [25]. Legal scholarship on digital technology in mental health care is sparse [1] but tends to focus on the regulatory frameworks applicable to digital health, privacy, confidentiality, cybersecurity, and software as medical devices [35-39].

The broader ethical and legal dimensions of algorithmic technologies have been the subject of a much larger scholarship [28-30,40,41]. Scholars in this field are typically concerned with issues of fairness, accountability, transparency, privacy, security, reliability, inclusivity, and safety, which are examined in contexts as diverse as criminal law, consumer transactions, health, public administration, migration, and employment. Legal scholars have tended to call for technological due process (involving fair, accountable, and transparent adjudications and rulemaking), net neutrality (broadly, equal treatment of web-based content by providers of internet access services) [42], and nondiscrimination principles [43]. Early legal and ethical scholarship focused on efforts to ensure basic standards of algorithmic transparency and auditing, but a more recent movement of scholars, regulators, and activists has begun to ask more fundamental questions, including whether algorithmic systems should be used at all in certain circumstances, and if so, who gets to govern them [44].

## Methods

### Design

This study adapted a scoping review methodology to undertake a broad exploration of the literature. Scoping reviews are particularly useful for surveying a potentially large and interdisciplinary field that has not yet been comprehensively reviewed and for which clarification of concepts is required [45], a characterization that appears apt for the use of algorithmic and data-driven technologies in mental health care. The scoping review method was also considered the most appropriate approach because it could capture the literature from several sources and disciplines with varying terminology and conceptual boundaries.

We adapted the Arksey and O’Malley framework for scoping reviews [46]. The framework involves the following five steps or framework stages: (1) identifying the research question; (2) identifying relevant studies; (3) selecting studies; (4) charting results; and (5) collating, summarizing, and reporting results.

A description of each step is outlined below.

We drew on elements of the Joanna Briggs Institute scoping review methodology [47] and PRISMA (Preferred Reporting Items for Systematic Reviews and Meta-analyses) extension for scoping reviews [48] to support the rigor of our methods. Study selection included all study types, and the overall aim was to chart data according to key issues, themes, and gaps [46]. Materials were analyzed using conventional content analysis supplemented with keyword-in-context analysis (discussed below).

### Identifying the Research Question (Step 1)

We sought to identify all studies within a selective sampling frame [49] that answered the following research questions:

- In what ways are algorithmic and data-driven technologies being used in the mental health context?
- How and to what extent are issues of law and ethics being addressed in these studies?

These questions were chosen to maintain a wide approach to generate the breadth of coverage [46].

### Identifying Relevant Studies (Step 2)

A rapid or streamlined literature search was conducted. We started with a search string that emerged from our initial literature review (noted in the *Background* section). However, the search string was updated as we surveyed the literature, and new terms and ideas from other disciplines and practices were considered. We also undertook a hand search of relevant reference lists of included papers to identify other papers for inclusion. The search was not exhaustive because of the breadth of the topic area, but it aimed to be inclusive of diverse disciplines and varying conceptualizations of the topic.

The following search strings emerged through an iterative process (Textbox 1). They were applied in keyword fields or abstract and title fields (where available in each database).

**Iteratively developed search string.**

**Scopus**

- (TITLE-ABS-KEY ('mental (health OR ill* OR disability OR impair*)' OR 'psychiatr*' OR 'psycholog*' OR 'beahvioral health') AND TITLE-ABS-KEY ('algorithm*' OR 'artificial intelligence' OR 'machine learning') AND TITLE-ABS-KEY ('internet' OR 'social media' OR 'chatbot' OR 'smartphone' OR 'tracking'))

**Embase Ovid**

- ('mental (health OR ill* OR disability OR impair*)' or 'psychiatr*' or 'beahvioral health').mp. [mp=title, abstract, heading word, drug trade name, original title, device manufacturer, drug manufacturer, device trade name, keyword, floating subheading word, candidate term word]
- “mental illness”.mp. or mental disease/
- algorithm/ or machine learning/ or artificial intelligence/
- ('algorithm*' or 'artificial intelligence' or 'machine learning').mp. [mp=title, abstract, heading word, drug trade name, original title, device manufacturer, drug manufacturer, device trade name, keyword, floating subheading word, candidate term word]
- Internet/ or “web-based”.mp.
- ('internet' or 'social media' or 'chatbot' or 'smartphone' or 'tracking').mp. [mp=title, abstract, heading word, drug trade name, original title, device manufacturer, drug manufacturer, device trade name, keyword, floating subheading word, candidate term word]
- The above search strings were applied in various combinations.

**Association for Computing Machinery**

- ('mental health' OR 'mental ill*' OR 'psychiatr*' OR 'behavio* health') AND ('algorithm*' OR 'artificial intelligence' OR 'machine learning') AND ('internet' OR 'social media' OR 'chatbot' OR 'smartphone' OR 'tracking')

No date limit was placed, although the search was conducted between August 2019 and February 2020 iteratively. A language filter was applied to focus on English-language results, which was applied for pragmatic reasons to reduce the search scope and complexity (for more on limitations, including terms we appear to have overlooked, see the *Discussion* section).

After an extensive search, 1078 relevant peer-reviewed research studies were identified in the study selection stage. From these, papers that were not available in English, duplicates, and papers not available in the full text were excluded.

### Study Selection (Step 3)

The process of identifying relevant studies among the 1078 papers was iterative, involving several discussions between coauthors. Unlike systematic reviews, where inclusion and exclusion criteria for studies are established at the outset, this study developed these criteria during the search process (Textbox 2) [46]. The purpose of deciding on criteria post hoc is to avoid barring studies that might not align with current understandings of the issue or topic [46]. This was especially important when including computer science databases in the search strategy because of the heterogeneity of studies broadly related to mental health.

**Inclusion and exclusion criteria.**

**Inclusion criteria**

- Study undertaken in a mental health context or with application to a mental health context
- Text available in English
- Study related broadly to the use of big data, internet technology, artificial intelligence, sensors, smart technology, and other contemporary algorithmic technologies

**Exclusion criteria**

- Commentary pieces
- Studies focused on other health conditions
- Application of data science methods to clinical data collected via clinical technologies (eg, application of data science methods to magnetic resonance imaging data)
- Data science methods paper with no specific real-world application or objective
- Application of data science methods to psychiatric research in general
- Studies applied to animals or animal models

**Figure 1:**
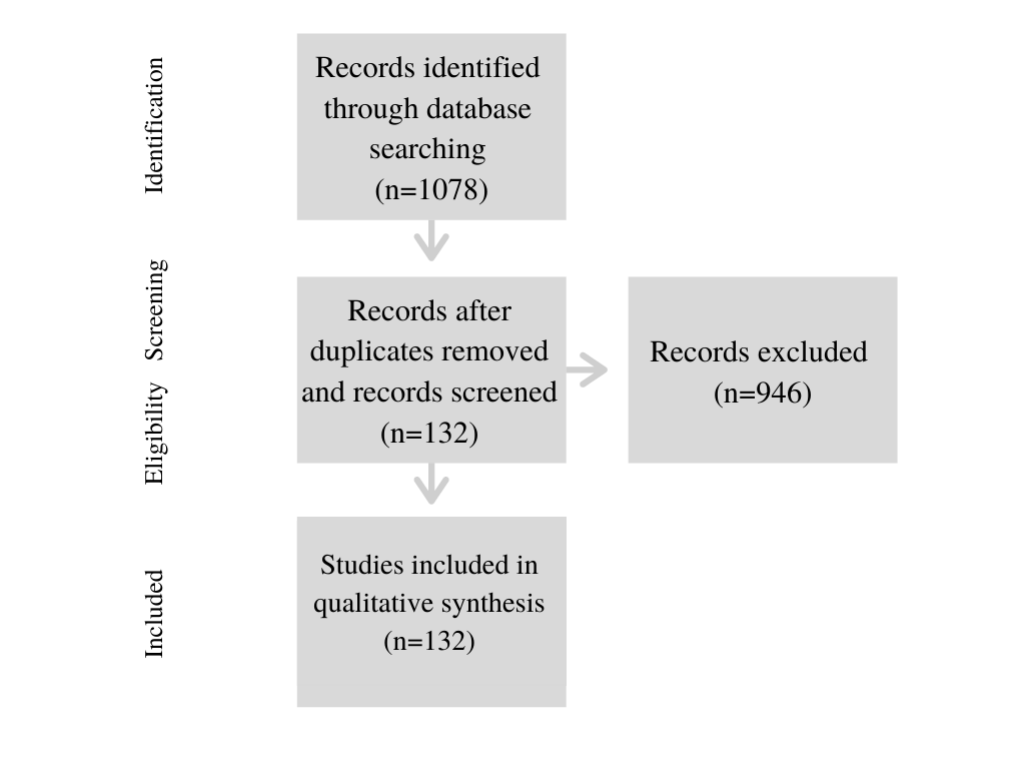
Study selection for review.

Owing to the large number of studies identified at step 2, we did not undertake a full-text review. Instead, we reviewed only the abstract and title according to our inclusion criteria. According to the PRISMA criteria described by Moher et al [50], systematic reviews would include a full-text review after duplicates were removed to assess all articles for eligibility. We did not take this step, as the screening and eligibility phases of the review could take place by reviewing the abstracts or titles (after all, we were simply looking for applied mental health research that used algorithmic and data-driven technologies—Figure 1).

This adaptation enabled us to review a large body of work in a rapidly expanding field. Our broad inclusion approach was also chosen to prevent the exclusion of studies from disciplines that do not conform to traditionally appropriate research designs, which might preclude them from reviews with stricter inclusion and exclusion criteria (eg, using an insufficient study design description as an exclusion criterion). For example, we found that many computer science papers were published in conference journals [51] and did not always include in-depth methods or an explicit statement of the research aim or objectives.

This process resulted in 132 empirical research papers included in the review. Figure 1 provides a PRISMA diagram that sets out the process of exclusion for our adapted study.

### Charting Results (Step 4)

Through initial deductive analysis of the abstracts and discussions between the researchers, we identified several key issues and themes through which to consider the broad research field. We settled on a typology that considered both the form of technology used in the study (eg, social media, sensors, or smartphones) and the stated purpose for the mental health initiative (eg, detection and diagnosis, prognosis, treatment, and support).

The second step involved analyzing the data to determine how legal and ethical issues were discussed. The material was analyzed using the computer software package NVivo 12 (QSR International) [52]. Conventional content analysis was undertaken, supplemented by keyword-in-context analysis [52]. We used the following terms, drawn from themes and keywords arising in the literature noted in the *Ethics and Law* section, which are typically associated with legal and ethical matters arising in the use of digital technologies in mental health: law* or legal*; ethic*; *human rights*; transparen*; *oversight*; accountab*; *bias*; *fairness*; *privacy*; *trust*; regulat*

We sought a uniform approach to the 132 studies included in this review. However, in practice, it was often impossible to extract all the information required where research reports used varying terminology and concepts and potentially failed to include relevant material.

### Collating, Summarizing, and Reporting Results (Step 5)

Several typologies can be used to categorize the algorithmic and data-driven technologies identified in these studies. As noted, we integrate two here: (1) the primary *forms* of technology and (2) their stated *purpose*. Such distinctions can help to highlight the predominant areas of technological inquiry and differentiate relevant ethical and legal concerns for the various categories.

## Results

### Typology: Form and Stated Purpose

#### Overview

We derived five major categories of technology (Textbox 3): (1) social media (53/132, 40.1%), (2) smartphones (37/132, 28%), (3) sensing technology (20/132, 15.1%), (4) chatbots (5/132, 3.8%), and (5) miscellaneous (17/132, 12.9%). We have discussed these categories in detail in the following sections. We further evaluated the papers according to the stated purpose of the technology using a typology created by Shatte et al [53]. They categorized papers into the following four categories: (1) detection and diagnosis; (2) prognosis, treatment, and support; (3) public health; and (4) research and clinical administration.

**Categorization of articles by the form and stated purpose of technology.**

**Social media**

- Detection and diagnosis (26/132, 19.7%) [54-79]
- Prognosis treatment and support (4/132, 3%) [80-83]
- Public health (22/132, 16.7%) [84-105]
- Research and clinical administration (1/132, 0.7%) [106]

**Smartphones**

- Detection and diagnosis (17/132, 12.9%) [107-123]
- Prognosis treatment and support (20/132, 15.1%) [124-143]
- Public health (0/132, 0%)
- Research and clinical administration (0/132, 0%)

**Sensing technology**

- Detection and diagnosis (6/132, 4.5%) [144-149]
- Prognosis treatment and support (12/132, 9.1%) [150-161]
- Public health (2/132, 1.5%) [162,163]
- Research and clinical administration (0/132, 0%)

**Chatbots**

- Detection and diagnosis (0/132, 0%)
- Prognosis treatment and support (5/132, 3.8%) [164-168]
- Public health (0/132, 0%)
- Research and clinical administration (0/132, 0%)

**Miscellaneous**

- Detection and diagnosis (8/132, 6.1%) [169-176]
- Prognosis treatment and support (8/132, 6.1%) [177-184]
- Public health (1/132, 0.7%) [185]
- Research and clinical administration (0/132, 0%)

Neat distinctions were not always possible. For example, Nambisan et al [95] sought to validate a method of detecting depression among social media users in a large-scale data set (a common aim in the social media category). At first glance, their study might appear to fall within the *detection and diagnosis* category. However, the ultimate aim of the study was to improve public health informatics, which improves the accuracy of population-wide prevalence analysis. Hence, we placed this study in the public health category (defined in the following sections).

The four categories by Shatte et al [53] offer clinical or medical framing, which broadly matches the clinical orientation of the scholarship (as a counterview, some researchers have called for the demedicalization of digital platforms designed to help people in mental distress [186], a point to which we will return in our discussion). Shatte et al [53] found that most studies in their scoping review on machine learning in mental health research focused on detection and diagnosis—this is indeed reflected in our own findings. We found that 43.2% (57/132) of the studies broadly concerned detection and diagnosis.

We determined that 37.1% (49/132) of the studies broadly concerned technology aimed primarily at prognosis, treatment, and support, which includes initiatives for personalized or tailored treatment and technologies used in services where treatment is provided. Examples include the use of smartphone apps to provide personalized education to someone based on psychometric data generated by the app.

A total of 18.9% (25/132) of studies were on public health. Public health papers used large epidemiological or public data sets (eg, social media data and usage data from Wi-Fi infrastructure) to monitor or respond to persons who appear to be experiencing or self-disclosing an experience of distress, mental health crisis, or treatment. However, we struggled in applying this category, as many were borderline cases in the detection and diagnosis category. This ambiguity may be because many studies were based in the field of computer science and were contemplated at a higher level of generality, with limited discussion of the specific setting in which they might be used (eg, a social media analytical tool could be used in population-wide prevalence studies or to identify and direct support to specific users of a particular web-based platform).

Our search uncovered only 1 study related to research and clinical administration; this particular study focused on the triage of patients in health care settings.

Finally, it is noteworthy that despite the reasonably large volume of studies, all but a few were at an exploratory and piloting stage. This is not surprising given the predominance in our survey of scholarship from computer science journals in databases such as ACM. A key issue in this area of inquiry is the large context gap between the design of these technological innovations and the context of implementation. In many papers from the computer science discipline, the authors made assumptions or guesses as to how their innovations could be implemented, with seemingly little input from end users. This is not a critique of individual researchers; instead, as we shall discuss later, it reflects the need for interdisciplinary and consultative forms of research at the early stages of ideation and piloting. This matter also raises questions as to whether there is a strong enough signal or feedback loop from practice settings back to designers and computer scientists in terms of what *needs* they should be responding to and why.

#### Social Media

We found 53 studies concerning social media, in which data were collected through social media platforms. Two major platform types were identified: mass social media, including mainstream platforms such as Facebook, Twitter, and Reddit; and specialized social media, comprising platforms focused on documenting health or mental health experiences. Both forms of social media involve the collection of textual data shared by users and self-reported mental health conditions, and sometimes expert opinion on diagnoses attributable to users (identified through information shared on the web). For example, researchers may examine whether the content of posts shared correlates with, and can therefore help predict, people’s self-reported mental health diagnosis. Most studies concerned mass social media platforms (Twitter: 17/53, 32%; Reddit: 14/53, 26%; Facebook: 6/53, 11%), with a small number concerning specialized social media (*PatientsLikeMe*: 1/53, 2%; *Psycho-babble*:1/53, 2%; *Reachout*: 1/53, 2%).

The largest sub-category in the social media group (26/53, 49%) have focused on predicting or detecting depression, with some concerning other diagnostic categories. Some studies attempted to capture multiple diagnostic categories or aimed to detect broad signs of mental ill-health.

#### Mobile Apps

In total, 38 studies concerned mobile apps used to collect and process data from participants, of which two main subcategories emerged. The first included apps that required active data input by participants (27/38, 71%), which either took the form of validated surveys (eg, Patient Health Questionnaire-9) or an experience sampling method; the second included those that passively collected data from inbuilt smartphone sensors (15/38, 39%). Some papers were counted twice as they had methods that covered both subcategories. Contemporary smartphones include a range of sensors related to sleep patterns, activity (movement), location data (GPS, Wi-Fi, and Bluetooth), communication or in-person human interaction (microphones), web-based activity (phone or text logs and app usage), and psychomotor data (typing and screen taps).

Apps that draw on these data sources can be considered passive sensing because the individual generally does not have to input data actively. Data collection generally requires participants to install an app that collects data from smartphone sensors and sends it to the researchers.

#### Sensing Technology

In total, 20 studies focused on broader sensor technology designed to continuously collect data on a person’s activity or environment. We differentiated this category from smartphone passive sensing, although there is a clear crossover with some personal wearables that fall under the sensing technology category. As we use it here, *sensing technology* includes a range of both wearables and environmental sensing technology (our search strings included variations on this theme, including *tracking*, *biometric monitoring*, and *behavioral sensing*). Many wearables were off-the-shelf personal wearables such as Fitbits, although there were several others, such as radio-frequency identification tags. Environmental sensors refer to technologies that collect data within the environment or about the environment but are not personal wearables, such as smart-home devices.

The list of sensing technologies includes personal wearables (9/20, 45%), smart-home sensors, automated home devices, internet of things (3/20, 15%), Microsoft Kinect (a software developer kit that includes computer vision, speech models, and algorithmic sensors; 1/20, 5%), skin conductance technology (1/20, 5%), portable electroencephalogram (1/20, 5%), radio-frequency identification tags (2/20, 10%), the use of Wi-Fi metadata (2/20, 10%), and data collected via care robots (eg, Paro Robot; 1/20, 5%).

Some studies have examined sensor systems for use in psychiatric settings. For example, Cheng et al [159] used a wireless monitoring system to monitor the location and heartrate of psychiatric inpatients. Other studies have used sensors in everyday settings. For example, Dickerson et al [158] sought to create a "real-time depression monitoring system for the home" for which data are collected that are "multi-modal, spanning a number of different behavioral domains including sleep, weight, activities of daily living, and speech prosody"

#### Chatbots

The fourth group of studies explored the use of chatbots and conversational agents in web-based mental health contexts, of which 5 studies appeared. This group includes studies focused on chatbots being used by both people experiencing mental health conditions or psychosocial disabilities and those who provide them with care or support. For example, D’Alfonso et al [83] studied the development of a "moderated online social therapy" web application, which provides an interactive social media-based platform for youth recovering from psychosis.

#### Miscellaneous

This final group (17/132, 12.9%) included a range of studies that did not fit the previous categories. This category included the collection of data from video games and data sources where there was no explicit outline of how such data would be collected in practice (eg, facial expression data). We included the video game data in this *miscellaneous* category, although it could also possibly sit in the social media category.

### Law and Ethics

#### Law

As noted, we conducted a thematic analysis supplemented by keyword-in-context analysis to identify themes related to law and ethics, as discussed in the *Background* section of this paper. There was little explicit discussion of legal issues, although issues such as privacy, which have precise legal dimensions, were discussed. However, privacy has rarely been discussed in terms of the law in the literature surveyed. We will return to the issue of privacy shortly. The term *law* appeared in just 1 study with reference to the legal implications of the particular algorithmic and data-driven technology being considered [187]. The term *legal* appeared in passing in three papers [92,99], among which the most substantial statement, by Faurholt-Jepson et al [133], referred to legal concerns as one of several considerations in different national contexts:

Using smartphones to collect large amounts of data on personal behavioral aspects leads to possible issues on privacy, security, storage of data, safety, legal and cultural differences between nations that all should be considered, addressed and reported accordingly.

133

A passing reference was made to the rights of user subjects in some studies (eg, Manikonda and De Choudhury [98] asked, “[h]ow...automated approaches, that are themselves prone to errors, [could] be made to act fairly, as well as secure one’s privacy, their rights on the platforms, and their freedom of speech?”). Other studies referred very briefly to compliance with the relevant regulatory or legislative frameworks under which the algorithmic and data-driven technologies were tested, such as the Health Insurance Portability and Accountability Act of 1996 (United States) [73,108,155].

#### Ethics

In terms of explicit reference to ethics, 10 studies included a specific section on the ethical issues raised by their work [64,73,80,87,92,98,99,105,163,187] and 10 others included a broad reference to key ethical issues [64,70,122,124,126,133,140,152,184]. The latter material varied from one or two sentences to a paragraph or more. Although we searched for several ethical and legal themes (eg, privacy, security, safety, transparency, autonomy, and justice), the theme of privacy was dominant.

#### Privacy

Privacy was discussed in several ways across all the included studies but was primarily addressed as part of the research method rather than in the real-world implementation of the technology. Approximately 19.7% (26/132) of papers sought to address user privacy through anonymization, deidentification, or paraphrasing of personal information. For example, Li et al [93] stated, “to protect Weibo users’ privacy, personally identiﬁable information (eg, names, usernames) were excluded from any research outputs.”

The second major approach concerns what we have referred to as *privacy protocols*. This included aligning processes to legal requirements [73,108,155,187] but for the most part concerned some kind of process for data management, such as ensuring the consent of and providing notice to user subjects. For example, Manikonda and De Choudhury [98] proposed *“*guidelines to be incorporated in the design and deployment of [their] interventions and tools,” which sought “voluntary consent from the population being studied and those likely to beneﬁt from the technologies.” However, unlike Manikonda and De Choudhury [98], very few studies have discussed the issue of consent at the implementation stage of their proposed technology. Instead, most authors discussed consent in terms of how their research was conducted. In some cases, mainly regarding social media, the authors argued that consent was not needed given the public nature of user-generated content on social media (a topic to which we will return).

A collection of privacy engineering approaches was taken, including hashing and encryption and managing the data processing location. There were a variety of approaches around when and where data were processed and how this aligned with ideas about privacy. Some studies have used encryption before sending data to servers for processing, whereas others have analyzed data locally on the smartphone or did not store specific data postprocessing. Wang et al [141], for example, noted, “we do not record any speech on the phone or upload to the cloud and all audio signal processing and feature extraction is based on privacy preserving algorithms.”

Some authors referred to the tension between privacy and data quality—framed, for example, as “[p]rivacy versus lives saved” [92]. This framing was seen within specific methods and technological approaches, such as the data and privacy-preserving qualities of sensor data, compared with Wi-Fi infrastructure data. For example, Ware et al [162] argued that whereas Wi-Fi infrastructure data could be considered more privacy-preserving than collecting data from smartphones, it may also be less accurate. Ji et al [66] discussed how a method they tested “has an advantage over data protection methods because it trains on the entire dataset, but it also violates user privacy and breaks the data protection setting,” and ultimately argued that their chosen “method achieves a balance between preserving privacy and accurate detection.”

The final point in the privacy theme was expectations. Very few studies have considered the expectations people may have about how their data are used. This led to an acknowledgment that the use of data from sources such as social media or video games to make predictions about people’s mental health changes the meaning of these data and could have unintended consequences. Eichstaedt et al [68], for example, noted that social media data used for health reasons might change how people perceive that data and thus the type of data they report. De Choudhury et al [99], in their analysis of *mental health content* in social media, warned that an unhelpful outcome could include “chilling effects in participation in the community, or suicide ideation moving on to fringe or peripheral platforms where such populations might be difficult to extend help to.”

## Discussion

### Principal Findings

#### Overview

To summarize, we identified five major types of technology—social media, mobile apps, sensing technology, chatbots, and others—in which algorithmic and data-driven technologies were applied in the mental health context. The primary stated purpose of these technologies was broadly to detect and diagnose mental health conditions (approximately 57/132, 43.2% of studies). Only 15.1% (20/132) of papers discussed ethical implications, with a primary focus on the individual privacy of research participants.

#### Privacy

As noted, the privacy of participants was addressed in the studies primarily with reference to engineering methods and, in some instances, concerning regulatory compliance. In the smartphone group, notice and consent combined with engineering methods were used to address user-subject privacy concerns. In the social media group, privacy was discussed in terms of how data were managed and the technical elements of the algorithms used, including privacy-preserving algorithms [96] and limiting the use of identifiable information [87]. In the sensor group, privacy was addressed in several ways, particularly by collecting low-fidelity data [150] and anonymization [148].

Questions may be raised about how privacy is (or should be) conceptualized and how the technologies will fare in real-world settings. Taking a strictly legal approach to privacy, for example, may not necessarily confer a social license to operate. An example of a failure to align law and social license is the United Kingdom’s proposed *care.data* scheme, where secondary data from general practitioners were to be collected for research purposes [188]. Although this scheme aligned, and in some cases, went further than legal requirements, it still faced a public backlash and was ultimately shut down.

Privacy as a concept exists as an expression of claims to dignity and self-determination. These more expansive concerns of dignity and autonomy were not the subject of explicit consideration in the studies examined in this review. This point raises the issue of possible gaps in the literature.

### Gaps

#### Overview

It is difficult to discuss what did *not* appear in the literature, as such observations are necessarily subjective and will differ based on a person’s disciplinary background, interests, and priorities. For our part, we noted four interconnected matters that we believe are important and which arise in the literature noted in the *Background* section. They are the paucity of ethical inquiry and consideration of algorithmic accountability, the near-complete lack of service user or subject input, and concerns with a medico-technological framing.

#### Gaps in Ethical Enquiry

Notwithstanding the common interest in matters of privacy across almost all papers, there was a relatively low engagement with broader ethical dimensions of the algorithmic and data-driven technology in question—a finding that appears to support the view of some scholars in the field [25].

However, an important distinction should be made between empirical studies designed to validate or explore a particular technology and (as we discussed in the *Background* section) the literature concerned *specifically* with ethical and legal issues arising from algorithmic technology. The very form of journal articles that examine applied research concerning algorithmic and data-driven technologies in mental health care may tend to preclude a focus on the ethical and legal issues that arise (although some authors clearly felt it worth noting pressing issues in their papers). Some disciplines, including computer science, appear to have traditionally separated ethics or legal articles from publications concerned with findings or validation regarding emerging technologies, although this tradition is somewhat challenged in the literature on ethics in design [189,190].

Furthermore, the gap between applied research, on the one hand, and research that is specifically focused on ethics, on the other hand, does not appear to be unique to the mental health context. For example, Hübner et al [191] point out that “ethical values have not yet found their firm place in empirically rigorous health technology evaluation studies” more generally. This dynamic “sets the stage for further research at the junction of clinical information systems and ethics” [191]. Indeed, others have sought to create frameworks to meet the new ethical and regulatory challenges of health care in the digital age [32].

A minority of the studies in our review discussed these challenges. Birnbaum et al [54], for example, discussed the limits of contemporary ethical standards for research on social media in the mental health context, noting that “[e]xisting ethical principles do not sufficiently guide researchers” and new technological approaches to “illness identification and symptom tracking will likely result in a redefinition of existing clinical rules and regulations.” However, many other studies have not discussed or alluded to these challenges. In one study, web-based videogame players were recruited to conduct a web-based survey asking for "sociodemographic and gaming information" and feedback concerning psychometric indicators to develop machine learning to predict psychological disorders. The researchers requested electronic consent from participants to take part in the study but “did not apply for, or receive, any approval from any board or committee for this research as this was a techno-behavioral general study which was non-medicinal, non-intrusive, and non-clinical in nature” [169]. Furthermore, the authors noted, “[we] are afﬁliated to a technology university which has no internal committee related to research on human subjects” [169].

New critical questions are required. For example, several studies in the social media category, the largest group of studies, eschewed institutional review board approval based on claims that their data sets were *publicly available*, raising ethical and legal concerns surrounding emergent, inferred, or indirect data concerning mental health and the potential appropriation of detection and screening tools in unethical (and potentially even illegal) ways. Such claims are being increasingly challenged, particularly following concerns about the creation of inferred data about unsuspecting and nonconsenting users in the health context generally [192] and the mental health context in particular [193]. Arguably, the likelihood of these risks being overlooked in research is exacerbated by the near-complete exclusion of persons with experience of mental health service use as active contributors to knowledge production in this field, whether as co- or lead investigators or even as advisors.

#### Lack of Service User Involvement

Very few studies (4/132, 3%) in this survey appear to have included people who have used mental health services, those who have experienced mental health conditions or psychosocial disability, or even those who were envisaged as end-beneficiaries of the particular algorithmic and data-driven technology, in the design, evaluation, or implementation of the proposals in any substantive way (except as research participants). In studies where service users were involved, this tended to comprise of research participants being involved in the co-design of content or codeveloping user-interfaces. D’Alfonso et al [83], for example, noted, “[t]he creation of therapy content [in their web-based platform]...was driven by feedback from users and expert youth mental health clinicians through iterative prototyping and participatory design.”

With very few exceptions, however, the survey indicated a near-complete exclusion of service users in the conceptualization or development of algorithmic and data-driven technologies and their application to mental health initiatives. It is also noteworthy that even mental health practitioners, who may well be end users envisaged by technologists, were involved in relatively few studies.

The active involvement of mental health service users and representative groups for persons with psychosocial disabilities has become a prominent ethos in mental health and disability policies worldwide [194] and is imperative in international human rights law [195]. A study in our survey included an acknowledgment of the limitations of not working with affected populations [105]. However, the authors referred to study populations as research subjects rather than active contributors to technological development. Manikonda and De Choudhury [98] did recommend the “[a]doption of user centered design approaches in intervention and technology development, to investigate specific needs and constraints of the target users, as well as their acceptability, utility, and interpretability.” Similarly, Ernala et al [73] noted that the field could benefit extensively from cross-disciplinary partnerships and partnerships between “computational and clinical researchers, and patients.” They also recommended “[p]articipatory research efforts such as the Connected and Open Research Ethics (CORE) initiative [for use] to develop dynamic and relevant ethical practices to guide and navigate the social and ethical complexities of patient data collection” [32,73].

From a pragmatic perspective alone, the involvement of service users and others with psychosocial disabilities is generally agreed to increase the likelihood of “viable and effective—rather than disruptive and short-lived—advances” in digital technologies in the mental health context [14].

Of the scant commentary and research in the field by persons with psychosocial disabilities and service users, commentators have raised concerns about: the potential need for a *right to explanation* concerning algorithmic decision making for individuals (not only the right of an individual to understand how a decision about them was made but also to query the values that go into a particular algorithmic decision system) [196]; the risk of discrimination or harm where sensitive personal information is leaked, stolen, sold, or scraped from social media [197]; and the deployment of data-driven technologies in coercive psychiatric interventions and policing [19,196]. Keyword searches along these lines did not yield any relevant results. Emerencia et al [184] prioritized the ethical imperative of shared decision making (“an approach in which patient and clinician are equal participants in deciding the treatment plan”) in their study on algorithmic technologies that might generate "personalized advice for schizophrenia patients", and Saha et al [87] highlighted the potential harms caused by the use of social media data to examine “psychopathological effects subject to self-reported usage of psychiatric medication.” However, these were unusual considerations among the studies reviewed and were noted in passing.

#### Concerns With Algorithmic Accountability

As discussed in the *Background* section, ethical and legal scholars on algorithmic and data-driven technologies have begun to raise fundamental concerns about whether algorithmic systems should be used at all for certain purposes and, if so, who should govern them [44]. Pasquale [44] illustrates the evolution of these concerns with reference to mental health apps:

For some researchers who are developing mental health apps, the first-wave algorithmic accountability concerns will focus on whether a linguistic corpus of stimuli and responses adequately covers diverse communities with distinct accents and modes of self-presentation. Second-wave critics...may bring in a more law and political economy approach, questioning whether the apps are prematurely disrupting markets for (and the profession of) mental health care in order to accelerate the substitution of cheap (if limited) software for more expensive, expert, and empathetic professionals.

Second-wave concerns give rise to questions as to who is benefiting from (and burdened by) data collection, analysis, and use [44]. Such concerns are spurred by questions about which systems deserve to be built, which problems most need to be addressed, and who is best placed to build and monitor them [198]. Scholarship on algorithmic and data-driven technologies in mental health services appears to have seldom asked such questions, at least explicitly ([196]; notable exceptions include [17] and [23]). The debate about algorithmic accountability in mental health care is likely to accelerate in the coming years amid broader calls for algorithmic decision systems to be subject to contest, account, and redress to citizens and representatives of the public interest.

#### Overmedicalization and Concerns of Techno-Solutionism

The issues the studies aimed to address were presented in medical terms and framed as problems that are amenable to digital technological solutions. This is not surprising. However, some scholars have raised concerns regarding this framing. In their survey of the messaging of mental health apps, Parker et al [186] argued that prominent apps tend to overmedicalize states of distress and may overemphasize “individual responsibility for mental well-being.” There may be legitimate reasons to demedicalize some approaches to supporting people in distress via digital initiatives and remain cautious about framing the matters as medical problems amenable to digital technological solutions [193,199]. Rose [194] argues that:

most forms of mental distress are inextricably linked to problems of poverty, precarity, violence, exclusion, and other forms of adversity in people’s personal and social experiences, and are best addressed not by medicalization, but by low intensity but committed and durable social interventions guided by outcomes that are not measured in terms of symptom reduction, but by the capacities that people themselves desire in their everyday lives.

This argument raises broader questions about the politics of mental health, for which it would be unrealistic to expect empirical studies of algorithmic and data-driven technologies in mental health care to resolve. Nevertheless, there is an argument that such political considerations and value choices are currently overlooked, with an overwhelming emphasis on scientific methods and measurements of risk and benefit.

### Comparison With Previous Work

Reviews such as those conducted by Shatte et al [53] and Tai et al [200] applied systematic literature search methods to identify the use of machine learning and artificial intelligence in modifying therapeutics and prevention strategies in psychiatry, and Doorn et al [201] performed a scoping review on its role in psychotherapy. However, to the best of our knowledge, no studies have surveyed the field to identify how ethical and legal issues are incorporated into applied research.

### Limitations

A disadvantage of using a rapid scoping review method is the difficulty in reproducing the results, given the use of numerous search strings in multiple combinations. This is exacerbated by our aim to cover multiple technology types across several cross-disciplinary databases (resulting in 1078 potential studies reduced manually to 132). There are trade-offs in this broad, exploratory approach. In addition to the challenges of replicability, we cannot claim to have achieved an exhaustive review, as may be possible in systematic reviews of specific technologies or subtypes (such as machine learning). Furthermore, the wide range of new and emerging technologies in our scope poses terminological challenges; hence, we undoubtedly missed studies that used terms overlooked in our search strings (as a peer reviewer pointed out, we did not use the term *recommender system*). This is exacerbated by the intrinsic challenge of pinning down terms and concepts in any area of rapid technological change [202].

Despite these limitations, a survey of empirical studies offers valuable information. The principal strength of a scoping review is its *breadth*. Our broad and cross-disciplinary approach enabled us to identify cross-cutting trends in the literature as a whole, and the trends we identified are striking, that is, roughly 15.1% (20/132) of the studies in the survey contained even a brief consideration of ethical issues, and only 3% (4/132) of studies appeared to involve mental health service users or affected populations. We argue that this is a significant finding that warrants our chosen method and research design.

### Conclusions

Our findings suggest that the disciplines undertaking applied research in this field do not generally prioritize *explicit* consideration of ethical and legal issues in their studies—and, perhaps more broadly, “the moral, political, social and policy issues at stake” [203]. Research institutions tend to focus strongly on protecting human participants involved in research, as they should, which is generally reflected in the studies in our survey (although not always). However, other important considerations, such as participatory and community-engaged research, which is an increasingly accepted requirement of mental health research, policy and practice, as well as broader ethicolegal issues in the field appear to be overlooked. This situation may have several explanations warranting further investigation, including editorial requirements for scholarly papers, the workings of institutional review mechanisms, funding arrangements, and prevailing evidentiary and epistemological cultures. However, with an increase in adverse effects involving flows of data concerning mental health [1], the situation must surely change.

## Abbreviations

PRISMA: Preferred Reporting Items for Systematic Reviews and Meta-analyses
